# Brazos Valley Medical Association

**Published:** 1896-12

**Authors:** H. W. Cummings

**Affiliations:** President; Hearne, Texas


### Society Notes.

### Proceedings of the Second Semi-Annual Meeting of the Brazos Valley Medical Association.

HEARNE, TEXAS, November, 1896.

The Brazos Valley Medical Association held their second semiannual meeting in Bryan, November 10th and 11th.

The meeting was called to order by the president, Dr. H. W. Cummings, of Hearne; and addresses of welcome were extended by Hon C. A. Adams, mayor, and Dr. Geo. R. Tabor. Response by the president.

The Judicial Council reported the following names as applicants for membership, who were elected:

Drs. H. L. Fountain, B. F. Watkins, R. H. Harrison and L. L. Todd, of Bryan; Drs. J. M. Soles, of Welborn; W. P. Gelstrap, of Wheelock; A. J. Adderhold, of Millican; J. P. Oliver, of Caldwell; John D. Porter, of Gause; A. G. Barnhill, of Sebesta; W. G. Peterson, of Navasota.

The regular programme was opened by a paper on "Brights' Disease" by Dr. J. M. Nicks, of Stone City, which was quite an exhaustive one and elicited much discussion.

Dr. Daniel Parker, of Calvert, read a very able paper on "Epithelioma," which was well received.

Next Dr. Geo. R. Tabor, of Bryan, read a paper on "Procidentia Uteri," which was tendered the association by Dr. Stoddard, Pueblo, Col., and which will appear in the *Southwestern Medical and Surgical Jonrnal* of Fort Worth.

Dr. I. P. Lessions, of Rockdale, presented a well written article on "Typho-Malarial Fever," and as he was unavoidably preveuted from attending, his paper was read by the secretary, and extensively discussed. By motion of the association, it was ordered published in the TEXAS MEDICAL JOURNAL.

The paper on "Appendicitis," by Dr. F. R. Collard, of Wheelock, was so elaborate that no one felt that he could add to it, so it received but little discussion. At the request of the *Southwestern Medical Record*, the association will send it for publication to that journal.

Quite a number of other papers not being sent in, the subjects were discussed by the members generally.

A committee of three members, one from each, Milam, Robertson and Brazos counties, and the president, were appointed to represent the association at the next meeting of the Texas Medical Association; and it is the desire of the B. V. M. A. to become an auxillary to same, and to present plans by which all smaller associations may become of much benefit to the State organization.

The association visited the A. & M. College, through invitation, and were highly entertained and pleasantly met by its Faculty.

Wednesday night a banquet was given to the doctors at the Exchange Hotel, at which many well-selected toasts were responded to.

The next meeting will be held the second Tuesday and Wednesday in May, 1897, at Cameron, Texas.

H. W. CUMMINGS, M. D., President.

